# Structural Validity and Measurement Properties of the Tampa Scale for Kinesiophobia in Knee Osteoarthritis: Comparison of Candidate Factor Structures

**DOI:** 10.1002/msc.70283

**Published:** 2026-10-12

**Authors:** José Djalma Arrais Junior, Lisa C. Carlesso, Barbara Greco Miura, Bruno Ruocco Verengue, Patrícia Gabrielle dos Santos, Almir Vieira Dibai‐Filho, Cid André Fidelis‐de‐Paula‐Gomes

**Affiliations:** ^1^ Postgraduate Program in Rehabilitation Sciences Nove de Julho University São Paulo São Paulo Brazil; ^2^ School of Rehabilitation Science McMaster University Hamilton Ontario Canada; ^3^ Postgraduate Program in Physical Education Universidade Federal do Maranhão São Luís Maranhão Brazil

**Keywords:** confirmatory factor analysis, knee osteoarthritis, measurement properties, outcomes research, psychometrics, structural validity

## Abstract

**Purpose:**

Kinesiophobia is clinically relevant in knee osteoarthritis (KOA), but the appropriate Tampa Scale for Kinesiophobia (TSK) structure remains uncertain. We compared candidate TSK factor structures and examined the selected measurement properties of the solution with the most favourable relative fit in this sample.

**Methods:**

Among 119 participants with KOA, ordinal confirmatory factor analysis tested three a priori structures: one‐factor 17‐item, one‐factor 11‐item, and two‐factor 11‐item. After three items in the two‐factor 11‐item structure had loadings below 0.20, a secondary exploratory 8‐item solution was examined. For this exploratory solution, convergent validity, test‐retest reliability, measurement error, composite reliability, and floor and ceiling effects were assessed.

**Results:**

All a priori structures had borderline fit and weak loadings. Among the models examined, the exploratory two‐factor 8‐item solution had the highest CFI and TLI (0.948 and 0.924), although its RMSEA was the highest (0.078). Reliability was substantial for Somatic focus (ICC = 0.85) and Activity avoidance (ICC = 0.78), with low measurement error. Composite reliability was adequate only for Activity avoidance.

**Conclusion:**

The exploratory 8‐item solution provided preliminary evidence, particularly for Activity avoidance, but requires independent validation.

## 1 Introduction

Knee osteoarthritis (KOA) is a leading cause of persistent pain, disability, and reduced participation among adults (Collins et al. 2026; Katz et al. 2021). Pain‐related fear may amplify disability through avoidance, deconditioning, and distress. Kinesiophobia, the excessive fear of movement arising from perceived vulnerability to pain or injury, is associated with pain, disability, and functional limitation in KOA (Kise et al. 2025; Lozano‐Meca et al. 2024).

In KOA, kinesiophobia has commonly been assessed using the original 17‐item Tampa Scale for Kinesiophobia (TSK‐17), with population‐specific evidence supporting its feasibility, reliability, and convergent validity (Dupuis et al. 2023; Areeudomwong and Buttagat 2017; Chua et al. 2025; Bruder et al. 2026). However, the optimal scoring structure of the TSK remains uncertain. A one‐domain structure assumes that all items reflect a single global construct of fear of movement, supporting interpretation via a total score. In contrast, two‐domain structures distinguish between activity avoidance, reflecting behavioural restriction related to movement or physical activity, and somatic focus, reflecting beliefs about bodily vulnerability and harm (Heuts et al. 2004; Roelofs et al. 2007). This distinction may be clinically relevant in KOA because avoidance behaviour and somatic threat appraisal may relate differently to pain, disability, physical activity, and psychological factors (Lozano‐Meca et al. 2024; Chua et al. 2025). Evidence for shorter TSK versions in KOA remains limited, but previous psychometric studies in musculoskeletal pain have reported heterogeneous item performance for the original TSK‐17 and have proposed reduced versions, including the TSK‐11, to improve interpretability and reduce item redundancy (Woby et al. 2005; Dupuis et al. 2023; Alpalhão et al. 2024). Therefore, direct comparisons of the original and reduced structures, as well as one‐ and two‐domain solutions, are needed before recommending a scoring approach for clinical stratification or research inference in individuals with KOA.

This study aimed to compare three candidate factor structures of the TSK in individuals with KOA and to provide preliminary evidence on selected measurement properties of the solution with the most favourable relative fit in this sample. Any reduced post hoc solution was treated as preliminary and hypothesis‐generating rather than definitive. Based on previous evidence of heterogeneous item performance in the TSK and prior support for reduced and two‐domain solutions in musculoskeletal pain and osteoarthritis, we hypothesised that reduced and/or two‐domain structures would show a more favourable relative fit than the original 17‐item, one‐domain structure in this KOA sample. Given the limited KOA‐specific evidence for shorter versions, this hypothesis was considered exploratory.

## 2 Methods

### 2.1 Study Design and Ethical Aspects

In accordance with the Declaration of Helsinki, this cross‐sectional study assessed the structural validity of three a priori candidate TSK factor structures in individuals with KOA and examined selected measurement properties of the secondary exploratory reduced solution, following the Consensus‐Based Standards for the Selection of Health Measurement Instruments (COSMIN) (Prinsen et al. 2018; Mokkink et al. 2010). The study was conducted from May 2023 to December 2024 at two physical therapy clinics and two primary care units in São Paulo, Brazil, and received approval from the institution's research ethics committee (Research Ethics Committee Opinion Number: 6.866.520, Certificate of Ethical Review Submission: 70415023.1.0000.5511). All participants provided written informed consent.

Potential participants were recruited sequentially through posters, brief presentations, waiting lists at the participating services, and in‐person or telephone invitations.

### 2.2 Sample Size

Sample size was planned using the conventional rule of at least seven participants per item; therefore, the 17‐item TSK required 119 participants (Terwee et al. 2012). This rule does not establish adequacy for ordinal CFA because requirements vary with model complexity, estimator, category distribution, and loading magnitude (Li 2016; Wolf et al. 2013). Accordingly, the analyses were treated as preliminary. The final 50 participants enrolled sequentially constituted the test‐retest subsample and were reassessed after seven days without treatment.

### 2.3 Participants and Eligibility Criteria

Eligible participants were men and women aged 40–75 years, literate in Brazilian Portuguese, with clinically diagnosed symptomatic KOA for at least three months, knee pain above 3 on the NPRS, morning stiffness under 30 minutes, crepitus, bony tenderness, and no palpable warmth (Verengue et al. 2025). An experienced musculoskeletal physical therapist confirmed the clinical diagnosis by physical examination. Radiographic confirmation and radiographic severity grading were not required.

Exclusion criteria were hip or lumbar symptoms that could confound knee‐specific kinesiophobia; other conditions that could mask KOA symptoms, including osteoporosis or fibromyalgia; cancer; active inflammatory joint disease; lower‐extremity joint replacement; neurological disease; or Mini‐Mental State Examination scores below education‐adjusted cutoffs (Murden et al. 1991; Verengue et al. 2025).

### 2.4 Measures, Instruments, and Procedures

Eligibility and clinical diagnosis were confirmed by an experienced musculoskeletal physical therapist. Four physical therapists, each with more than 10 years of experience, administered the assessments after 10 hours of standardized training. Participants completed the questionnaires independently in a private room without a time limit. An assessor was available only for procedural clarification and was instructed not to interpret items, suggest answers, or provide response‐influencing examples. The assessments followed a fixed sequence.

Demographic data, anthropometric data (body mass and height), and symptom duration (years since symptom onset) were recorded. Pain intensity during the previous seven days was measured using the 0‐to‐10 Numeric Pain Rating Scale (Costa et al. 2008). Pain catastrophizing was measured with the 13‐item Pain Catastrophizing Scale, which yields rumination, magnification, and helplessness scores (Ong et al. 2021; Sehn et al. 2012). Disability was measured with the 12‐item WHODAS 2.0 using simple scoring (range 12–60; higher scores indicate greater disability) (Kutlay et al. 2011; Silva et al. 2013).

The TSK‐17 is a 17‐item instrument translated into Brazilian Portuguese that measures fear of movement. Items 4, 8, 12, and 16 are reverse‐scored. Response options range from “strongly disagree” to “strongly agree,” and total scores range from 17 to 68. Higher scores indicate greater maladaptive beliefs about movement (Siqueira et al. 2007; Dupuis et al. 2023). TSK was administered on a single occasion and only in its original 17‐item form. Because the 11‐item TSK is a subset of the original instrument, comprising items 1, 2, 3, 5, 6, 7, 10, 11, 13, 15, and 17, the 11‐item scores were obtained from that same administration, and no additional TSK form was completed by participants. The shortened 11‐item version of the TSK was developed from the original TSK‐17 and has shown acceptable psychometric properties in chronic pain and musculoskeletal pain populations (Woby et al. 2005; Alpalhão et al. 2024).

Three a priori TSK factor structures were evaluated. Structure 1 was the original 17‐item TSK modelled as a single domain (Siqueira et al. 2007; Dupuis et al. 2023). Structures 2 and 3 were based on the same 11 items obtained from a single administration. Both structures contained identical items and differed only in the scoring structure imposed in the confirmatory factor analysis. Structure 2 modelled the 11 items as a single domain, representing a global fear of movement score (Woby et al. 2005; Alpalhão et al. 2024). Structure 3 modelled the same 11 items as two correlated domains, consistent with previously proposed two‐domain TSK structures. The Somatic focus domain included items 3, 5, 6, 7, and 11, and the Activity avoidance domain included items 1, 2, 10, 13, 15, and 17 (Heuts et al. 2004; Roelofs et al. 2007). All structures were fitted to the same participant responses, so no item was answered more than once. Structural validity was examined for these three a priori structures, whereas the measurement properties reported below were estimated only for the secondary exploratory reduced solution described in the Results.

### 2.5 Statistical Analysis

Continuous variables were summarised as means and standard deviations, and categorical variables as frequencies and percentages. Data completeness was checked before analysis. All 119 participants had complete baseline analysis data, and all 50 retest participants had complete retest data; no imputation was required.

Ordinal CFA was conducted in R (version 4.5.2; R Core Team 2025) with lavaan (version 0.6.21; Rosseel 2012) and semPlot (version 1.1.7; Epskamp 2015). Items were declared ordered, and models were estimated from polychoric correlations with WLSMV (diagonally weighted least squares with robust standard errors and a mean‐ and variance‐adjusted test statistic). Fit was assessed with CFI, TLI, RMSEA, SRMR, and chi‐square/df; CFI and TLI > 0.90, RMSEA and SRMR < 0.08, and chi‐square/df < 3 were the prespecified criteria (Schermelleh‐Engel et al. 2003). SRMR was prespecified but was not extracted for the final models and is therefore not reported; fit was judged on the remaining indices. AIC and SABIC are not defined under DWLS estimation; they were therefore obtained from maximum likelihood fits of the same models with items treated as continuous and are reported for transparency only. Because information criteria are most appropriate for comparing models fitted to the same observed variables, AIC and SABIC were interpreted cautiously. These indices were considered directly comparable only for structures based on the same item set, particularly Structures 2 and 3, which used the same 11 items. For comparisons involving structures with different observed item sets, including the 17‐item structure and the exploratory 8‐item reduced solution, model interpretation relied primarily on global fit indices, standardized factor loadings, and theoretical coherence.

Convergent validity was evaluated with Spearman's rho, with all correlations expected to be positive. For Activity avoidance, at least moderate correlations were expected with pain and disability and weak correlations with the three PCS domains. For Somatic focus, moderate correlations were expected with PCS rumination and helplessness, a weak‐to‐moderate correlation with PCS magnification, and weak correlations with pain and disability. Correlations were weak below 0.30, moderate from 0.30 to 0.59, and strong at 0.60 or higher. A hypothesis was supported when direction and magnitude met or exceeded the prespecified category; convergent validity required confirmation of at least 75% of hypotheses (Mokkink et al. 2018; Terwee et al. 2012).

Internal consistency was assessed using composite reliability (CR), with values ≥ 0.70 considered adequate (Raykov 1997). We used the online calculator from The Statistical Mind available at https://thestatisticalmind.com/comp‐reliability/.

Test‐retest reliability was assessed after 7 days without treatment, an interval intended to reduce recall while limiting clinical change. ICC(2,1), 95% CI, SEM, and MDC were calculated. ICC values were interpreted as low (< 0.40), moderate (0.40–0.75), substantial (0.75–0.90), or excellent (> 0.90). SEM percentages were very good (≤ 5%), good (> 5%–10%), questionable (> 10%–20%), or poor (> 20%). SEM = SD × √(1 − ICC) and MDC = 1.96 × SEM × √2 (Terwee et al. 2012; Barreto et al. 2022).

Floor and ceiling effects were evaluated. These effects were deemed present when 15% or more of the sample reached the minimum or maximum score of either domain of the 8‐item solution (Terwee et al. 2012).

## 3 Results

### 3.1 Participant Characteristics

Of 131 individuals assessed for eligibility, 12 were excluded: 4 had fibromyalgia, 2 had active inflammatory joint disease, and 6 did not have a confirmed KOA diagnosis. The remaining 119 were included; all baseline data and all data from the 50 retest participants were complete. Mean age was 60.93 (SD 9.32) years, mean BMI was 28.26 (SD 4.75) kg/m^2^, and 93 participants (78.2%) were women. Mean symptom duration was 8.15 (SD 2.63) years and mean pain intensity was 4.22 (SD 0.85) on the NPRS (Table 1).

**TABLE 1:** Characteristics of the study participants (*n* = 119).

Variables	*n* (%) or mean (SD)
Age (years)	60.93 (9.32)
Body mass (kg)	76.34 (11.81)
Height (m)	1.64 (0.09)
BMI (kg/m^2^)	28.26 (4.75)
Sex	
Male	26 (21.8)
Female	93 (78.2)
Marital status	
Single	10 (8.4)
Married	61 (51.3)
Widowed	21 (17.6)
Divorced	27 (22.7)
Education	
University complete	41 (34.5)
High school complete	57 (47.9)
Elementary school complete	21 (17.6)
Affected knee	
Right	47 (39.5)
Left	34 (28.6)
Bilateral	38 (31.9)
Symptom duration (years)	8.15 (2.63)
TSK‐17 (17–68 points)	39.40 (5.12)
TSK‐11 (11–44 points)	23.46 (4.06)
NPRS (0–10 points)	4.22 (0.85)
PCS	
Rumination (0–16 points)	10.44 (1.70)
Magnification (0–12 points)	7.02 (1.05)
Helplessness (0–24 points)	10.47 (1.79)
WHODAS 2.0 (12–60 points)	24.02 (2.51)

*Note:* Data are *n* (%) or mean (SD). The TSK‐11 score was derived from the same TSK‐17 administration and was not collected through a separate questionnaire.

Abbreviations: BMI = body mass index; kg = kilogramme; m = metre; NPRS = Numeric Pain Rating Scale (higher score = greater pain intensity); PCS = Pain Catastrophizing Scale (higher score = greater catastrophizing); SD = standard deviation; TSK‐11 = 11‐item Tampa Scale for Kinesiophobia (higher scores = greater kinesiophobia); TSK‐17 = 17‐item Tampa Scale for Kinesiophobia; WHODAS 2.0 = 12‐item World Health Organisation Disability Assessment Schedule 2.0, simple scoring (higher score = greater disability).

We first tested three a priori candidate TSK structures in individuals with KOA: the original 17‐item one‐domain structure, the 11‐item one‐domain structure, and the 11‐item two‐domain structure. These three a priori structures showed borderline fit indices and several weak item loadings. The secondary exploratory reduction was derived from Structure 3 for both statistical and theoretical reasons. Statistically, Structure 3 showed relative fit comparable to the 11‐item one‐domain structure, with the same AIC and a slightly lower SABIC. Theoretically, Structure 3 was the only a priori structure that preserved the distinction between Somatic focus and Activity avoidance, allowing us to examine whether this clinically relevant two‐domain interpretation could be retained after removing poorly performing items. In Structure 3, items 5, 10, and 13 showed factor loadings below 0.20 and were therefore removed. This resulted in an 8‐item two‐domain structure, with Somatic focus represented by items 3, 6, 7, and 11, and Activity avoidance represented by items 1, 2, 15, and 17.

Among the models examined, the secondary reduced 8‐item two‐domain solution (Structure 4) had the highest CFI and TLI values, whereas its RMSEA and chi‐square/df were higher than those of the a priori structures; its lower AIC and SABIC reflect fewer observed items and do not indicate better fit (Table 2). Because this solution was derived and evaluated in the same dataset and retained weak loadings (Table 3), this advantage is relative to the models examined and does not demonstrate adequate structural validity.

**TABLE 2:** Fit indices of the tested Tampa Scale for Kinesiophobia factor structures in individuals with knee osteoarthritis.

Fit index	Structure 1	Structure 2	Structure 3	Structure 4
*χ* ^2^/df	1.24	1.45	1.47	1.71
CFI	0.906	0.919	0.918	0.948
TLI	0.892	0.899	0.895	0.924
RMSEA (90% CI)	0.045 (0.011–0.067)	0.062 (0.022–0.093)	0.063 (0.023–0.095)	0.078 (0.026–0.112)
AIC	4557.802	3024.699	3024.699	2173.439
SABIC	4544.805	3016.289	3015.907	2166.940

*Note:* *χ* ^2^/df, CFI, TLI, and RMSEA were obtained with WLSMV estimation. AIC and SABIC were obtained from maximum likelihood fits with items treated as continuous and are directly comparable only between Structures 2 and 3, which share the same items. SRMR was prespecified but is not reported (see Statistical analysis). Structure 1: One domain and 17 items; Structure 2: One domain and 11 items; Structure 3: Two domains and 11 items (Somatic focus domain: items 3, 5–7, 11; Activity avoidance domain: items 1, 2, 10, 13, 15, 17); Structure 4: Two domains and 8 items (Somatic focus domain: items 3, 6, 7, 11; Activity avoidance domain: items 1, 2, 15, 17). Structure 4 was a secondary exploratory post hoc reduced solution derived from Structure 3 after excluding items with factor loadings below 0.20.

Abbreviations: AIC = Akaike information criterion; CFI = comparative fit index; CI = confidence interval; df = degrees of freedom; RMSEA = root mean square error of approximation; SABIC = sample‐size adjusted Bayesian information criterion; TLI = Tucker‐Lewis index.

**TABLE 3:** Factor loadings of the tested Tampa Scale for Kinesiophobia factor structures in individuals with knee osteoarthritis.

Item	Structure 1	Structure 2	Structure 3	Structure 4
Item 1	0.803	0.780	0.785	0.780
Item 2	0.657	0.684	0.692	0.691
Item 3	0.480	0.628	0.684	0.684
Item 4	0.449	—	—	—
Item 5	0.133^a^	0.095^a^	0.107^a^	—
Item 6	0.383	0.347	0.367	0.347
Item 7	0.515	0.552	0.592	0.588
Item 8	0.395	—	—	—
Item 9	−0.089^a^	—	—	—
Item 10	−0.032^a^	−0.061^a^	−0.060^a^	—
Item 11	0.326	0.312	0.324	0.321
Item 12	0.033^a^	—	—	—
Item 13	−0.021^a^	0.075^a^	0.079^a^	—
Item 14	0.125^a^	—	—	—
Item 15	0.170^a^	0.235	0.238	0.244
Item 16	0.546	—	—	—
Item 17	0.687	0.687	0.700	0.703

*Note:* Structure 1: One domain and 17 items; Structure 2: One domain and 11 items; Structure 3: Two domains and 11 items (Somatic focus domain: items 3, 5–7, 11; Activity avoidance domain: items 1, 2, 10, 13, 15, 17); Structure 4: Two domains and 8 items (Somatic focus domain: items 3, 6, 7, 11; Activity avoidance domain: items 1, 2, 15, 17).

^a^ Factor loading below the 0.20 screening threshold.

In Structure 4, all retained items had factor loadings above the prespecified exclusion threshold of 0.20. However, some retained items still showed weak loadings, particularly item 15 in the Activity avoidance domain and items 6 and 11 in the Somatic focus domain. Therefore, these items should be interpreted as relatively weak indicators of their respective latent domains, and the reduced solution should be considered preliminary. Loadings ranged from 0.24 to 0.78 in Activity avoidance and from 0.32 to 0.68 in Somatic focus (Table 3). Figure 1 presents the path diagram of this model, including the standardized coefficients for each domain and item.

**FIGURE 1:**
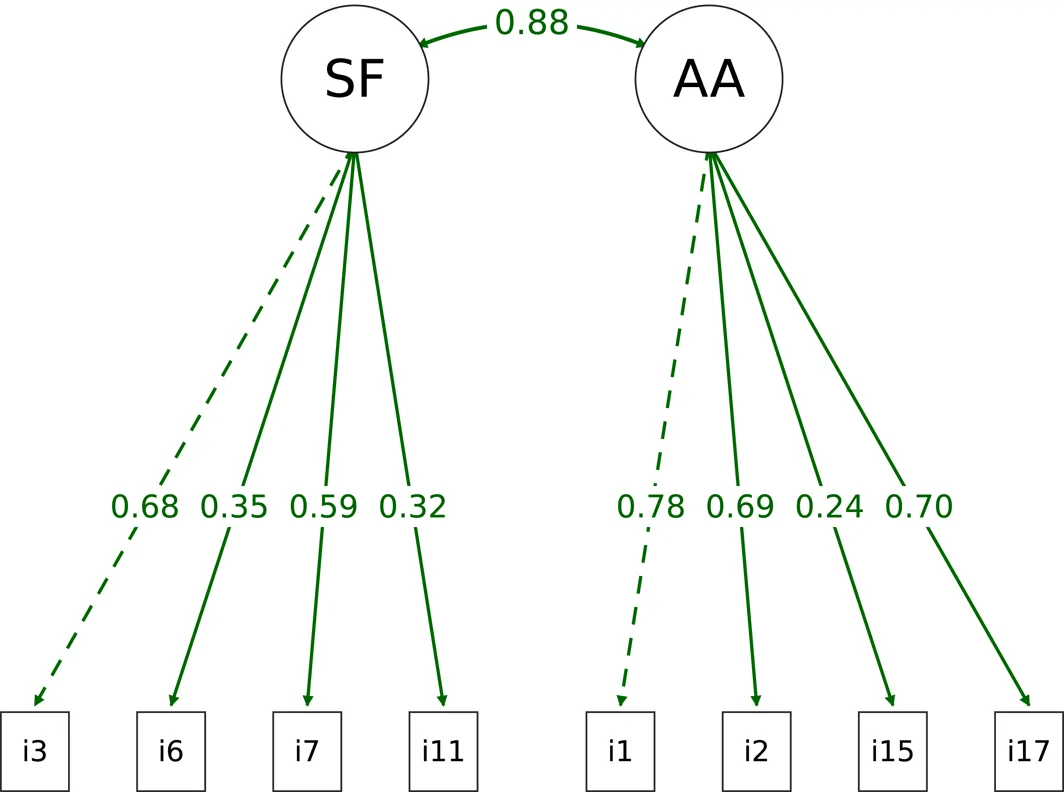
Path diagram of the reduced two‐factor, 8‐item solution of the Tampa Scale for Kinesiophobia in individuals with knee osteoarthritis. Circles represent latent domains, and rectangles represent observed items. Solid single‐headed arrows denote freely estimated factor loadings, whereas dashed single‐headed arrows identify the marker‐variable loadings fixed for model identification in the unstandardised solution; all displayed loading values are standardized. Double‐headed arrow shows the standardized correlation between the two latent domains. Somatic focus includes items 3, 6, 7, and 11. Activity avoidance includes items 1, 2, 15, and 17.

All measurement properties reported in this section refer exclusively to the secondary exploratory reduced two‐domain 8‐item solution (Structure 4). Convergent validity, test‐retest reliability, measurement error, composite reliability, and floor and ceiling effects were not estimated for Structures 1, 2, or 3. Because this solution remains preliminary, the measurement properties described below should be read as preliminary evidence.

The convergent validity hypotheses were partially supported. Several correlations were in the expected positive direction, but some associations were weaker than hypothesised, particularly the correlations between Activity avoidance and disability and between Somatic focus and PCS helplessness. For the Somatic focus domain, correlations were weak with pain intensity (rho = 0.16), disability (rho = 0.22), and PCS magnification (rho = 0.29), moderate with PCS rumination (rho = 0.39), and weak with PCS helplessness (rho = 0.11). For the Activity avoidance domain, correlations were strong with pain intensity (rho = 0.63) but weak with disability (rho = 0.25), PCS rumination (rho = 0.29), PCS magnification (rho = 0.28), and PCS helplessness (rho = 0.13) (Table 4).

**TABLE 4:** Convergent validity, test‐retest reliability, measurement error, and composite reliability of the exploratory two‐domain, 8‐item solution (Structure 4) of the Tampa Scale for Kinesiophobia in individuals with knee osteoarthritis (Somatic focus: items 3, 6, 7, and 11; Activity avoidance: items 1, 2, 15, and 17).

	Somatic focus domain		Activity avoidance domain	
Properties	Values	Classification	Values	Classification
Convergent validity (*n* = 119)				
NPRS (0–10 points)	0.16	Weak	0.63**	Strong
PCS				
Rumination (0–16 points)	0.39**	Moderate	0.29**	Weak
Magnification (0–12 points)	0.29**	Weak	0.28**	Weak
Helplessness (0–24 points)	0.11	Weak	0.13	Weak
WHODAS 2.0 (12–60 points)	0.22*	Weak	0.25**	Weak
Test‐retest reliability (*n* = 50)				
Mean (SD)‐1	10.24 (1.23)		10.64 (1.25)	
Mean (SD)‐2	9.82 (1.20)		10.38 (1.39)	
ICC (95% CI)	0.85 (0.75–0.91)	Substantial	0.78 (0.62–0.88)	Substantial
SEM (points)	0.47	—	0.62	—
SEM (%)	4.71%	Very good	5.89%	Good
MDC (points)	1.31		1.72	
MDC (%)	13.05%	—	16.33%	—
Composite reliability	0.56	Inadequate	0.71	Adequate

*Note:* Convergent validity values are Spearman's rho.

Abbreviations: CI = confidence interval; ICC = intraclass correlation coefficient; MDC = minimal detectable change; NPRS = Numeric Pain Rating Scale (higher score = greater pain intensity); PCS = Pain Catastrophizing Scale; SEM = standard error of measurement; WHODAS 2.0 = 12‐item World Health Organization Disability Assessment Schedule 2.0, simple scoring (higher score = greater disability).

^*^ *p* < 0.05.

^**^ *p* < 0.01.

In the test‐retest subsample (*n* = 50), reliability was substantial for the Somatic focus domain (ICC = 0.85, 95% CI 0.75–0.91) and the Activity avoidance domain (ICC = 0.78, 95% CI 0.62–0.88). Measurement error was low for both domains. The SEM was 0.47 points for Somatic focus and 0.62 points for Activity avoidance, corresponding to SEM percentages of 4.71% and 5.89%, respectively. The MDC was 1.31 points for Somatic focus and 1.72 points for Activity avoidance, corresponding to MDC percentages of 13.05% and 16.33%, respectively.

Composite reliability was inadequate for the Somatic focus domain (0.56) and adequate for the Activity avoidance domain (0.71), suggesting that domain‐level interpretation should be more cautious for the Somatic focus domain.

No floor or ceiling effects were observed in either domain. For Somatic focus, 7 participants (5.9%) achieved the minimum score and 2 (1.7%) achieved the maximum score. For Activity avoidance, 3 participants (2.5%) achieved the minimum score and 1 (0.8%) achieved the maximum score.

## 4 Discussion

This study compared three a priori candidate factor structures of the TSK in individuals with KOA and found that a secondary exploratory reduced two‐domain solution, comprising items 1, 2, 3, 6, 7, 11, 15, and 17, had the highest CFI and TLI among the examined structures. This advantage was confined to incremental fit because its RMSEA was the highest of the four models and the upper limit of its 90% CI exceeded 0.08. Therefore, it reflects relative fit within the models examined and not adequate structural validity. However, because it was derived after item removal within the same dataset, it should be viewed as preliminary, exploratory, and hypothesis‐generating rather than confirmed.

The hypotheses were partially supported. The reduced two‐domain solution had the most favourable incremental fit among the models examined, although the RMSEA confidence interval indicated residual uncertainty about model fit. Convergent validity hypotheses were also partially supported, though some associations were weaker than expected. In addition, composite reliability was adequate only for Activity avoidance. These findings suggest that the selected structure should be interpreted cautiously, particularly for the Somatic focus domain.

Within the models examined, Structure 4 showed the most favourable relative performance based on incremental fit, loadings, and theoretical coherence. Its lower AIC and SABIC were not decisive because it contained fewer observed items. As this two‐factor solution was derived after removing items below 0.20, it remains preliminary and requires independent validation.

Loadings of approximately 0.20–0.35 indicated that items 15, 6, and 11 were weak indicators. They were retained because they exceeded the 0.20 screening rule and further deletion in the same sample would increase overfitting and reduce content coverage, leaving Somatic focus with too few indicators. No further deletion was undertaken. Future work should use independent exploratory and confirmatory samples, Rasch analysis, item response theory, or cross‐validation before proposing a definitive KOA version.

The 0.20 rule was an investigator‐defined, deliberately permissive screening threshold intended to remove only items with near‐null loadings while limiting additional data‐driven deletion and loss of content. It is not a COSMIN criterion or a universally accepted cutoff. The reduced solution is therefore conditional on this analytic choice.

Prior KOA evidence also supports two TSK factors, Activity avoidance and Somatic focus (Heuts et al. 2004). The present item composition differed, indicating that item behaviour may vary across populations and that the exploratory structure should be tested independently before domain‐level scoring is adopted.

Activity avoidance was strongly associated with pain intensity, whereas Somatic focus was moderately related to rumination and weakly related to pain. This domain difference is consistent with fear‐avoidance theory (Vlaeyen and Linton 2000) and contrasts with broader correlations reported for total TSK‐17 scores in Thai KOA (Areeudomwong and Buttagat 2017). Because several prespecified correlations were weaker than expected, the findings provide only partial preliminary convergent‐validity evidence.

Composite reliability was adequate for Activity avoidance (0.71) but inadequate for Somatic focus (0.56), consistent with the weaker loadings of items 6 and 11. A similar domain asymmetry was reported in OA by Heuts et al. (2004), although the present exploratory domain scores require independent confirmation.

Domain scoring may distinguish Activity avoidance from Somatic focus, but this interpretation remains preliminary because Somatic focus had lower composite reliability and the reduced solution lacks independent validation.

This study has several limitations. The sample was recruited from health services rather than the community, which may limit the generalisability of the results to the broader KOA population. Generalisability may also be limited by the language used to administer the measures, since the TSK was administered in Brazilian Portuguese, and slight differences between translated versions may influence item interpretation and factor loadings. Another limitation is that neither radiographic confirmation nor radiographic severity grading was required. Therefore, structural severity could not be characterised, and we could not examine whether radiographic severity influenced pain, disability, or fear‐related beliefs in this sample. Additionally, although the sample size was calculated using a conventional item‐to‐participant ratio, 119 participants may still be insufficient for ordinal confirmatory factor analysis, particularly because multiple competing factor structures were examined. This may have reduced the precision of parameter estimates, affected the stability of factor loadings, and increased uncertainty around the reduced 8‐item solution.

Although recruitment also included two primary care units, all four sites were in one city and participants were already engaged with health services; therefore, they may have had greater symptom burden than community samples. The predominance of women (78.2%) limits sex‐specific inference. Measurement invariance across sex, age, education, pain severity, and language was not tested. With only 26 men and still smaller strata for other characteristics, post hoc ordinal multigroup CFA would not have produced stable subgroup estimates; comparisons across these groups are therefore not supported.

The retained items and apparent fit should be interpreted cautiously. The 8‐item solution was developed and tested in the same sample, creating an overfitting and potentially optimistic fit. It requires validation in a larger independent KOA sample before clinical or research use. Measurement invariance and responsiveness were not assessed. No stability anchor confirmed symptom stability at retest, so reliability may partly reflect true change. Future studies should use larger, diverse, multicenter samples, test responsiveness and meaningful change, include a retest stability anchor, and examine item performance with Rasch, item response theory, alternative item sets, additional reliability coefficients, and differential item functioning. Accordingly, 119 participants should not be regarded as adequate for definitive ordinal CFA or multiple model comparisons, and the fit indices and factor loadings may be unstable (Li 2016; Wolf et al. 2013). Although questionnaire completion was self‐reported and procedural assistance was standardized, assessor presence may still have influenced responses; its direction and magnitude cannot be estimated. Absolute fit could not be evaluated with SRMR.

## 5 Conclusions

Among the tested TSK structures in individuals with KOA, a secondary exploratory reduced two‐factor solution with 8 items (1, 2, 3, 6, 7, 11, 15, and 17) showed the most favourable relative fit among the models examined in this sample, which does not establish adequate structural validity. Both domains demonstrated substantial test‐retest reliability and low measurement error, with standard errors of measurement corresponding to 4.71% and 5.89% of the observed domain scores. Composite reliability was adequate for Activity avoidance but not for Somatic focus, indicating greater confidence in interpreting Activity avoidance and more caution with Somatic focus. Because this reduced solution was developed and tested within a single sample, it should be considered preliminary and hypothesis‐generating, and requires external validation in independent KOA samples before wider domain‐based use.

## Author Contributions

José Djalma Arrais Junior, Lisa C. Carlesso, Almir Vieira Dibai‐Filho, and Cid André Fidelis‐de‐Paula‐Gomes contributed to the conception and design of the study. Barbara Greco Miura, Bruno Ruocco Verengue, and Patrícia Gabrielle dos Santos contributed to data collection. José Djalma Arrais Junior and Cid André Fidelis‐de‐Paula‐Gomes performed the statistical analysis. José Djalma Arrais Junior, Lisa C. Carlesso, and Cid André Fidelis‐de‐Paula‐Gomes drafted the manuscript. All authors critically revised the manuscript for important intellectual content and approved the final version.

## Funding

This work was partially supported by Coordenação de Aperfeiçoamento de Pessoal de Nível Superior (CAPES), finance code 001.

## Ethics Statement

This study followed relevant international guidelines and regulations, and the methods were approved by the Research Ethics Committee of Nove de Julho University (Research Ethics Committee Opinion Number: 6.866.520; Certificate of Ethical Review Submission: 70415023.1.0000.5511). All study participants signed an informed consent form.

## Consent

Informed consent was obtained from all the individual participants in the study. Patients provided informed consent to publish their data.

## Conflicts of Interest

The authors declare no conflicts of interest.

## Data Availability

The datasets generated and/or analyzed during the current study are not publicly available because they contain information that could compromise participant privacy and are subject to institutional and ethical restrictions. Deidentified data may be made available by the corresponding author on reasonable request, subject to approval by the relevant ethics committee and institutional requirements.
